# Understanding the temporal evolution of neuronal connectivity in cultured networks using statistical analysis

**DOI:** 10.1186/1471-2202-15-17

**Published:** 2014-01-21

**Authors:** Alessandro Napoli, Jichun Xie, Iyad Obeid

**Affiliations:** 1 Department of Electrical and Computer Engineering, Temple University, Philadelphia, PA, USA; 2 Department of Statistics, Fox School of Business, Temple University, Philadelphia, PA, USA

## Abstract

**Background:**

Micro-Electrode Array (MEA) technology allows researchers to perform long-term non-invasive neuronal recordings in-vitro while actively interacting with the cultured neurons. Despite numerous studies carried out using MEAs, many functional, chemical and structural mechanisms of how dissociated cortical neurons develop and respond to external stimuli are not yet well understood because of the lack of quantitative studies that assess how their development can be affected by chronic external stimulation.

**Methods:**

To investigate network changes, we analyzed a large MEA data set composed of neuron spikes recorded from cultures of dissociated rat cortical neurons plated on MEA dishes with 59 recording electrodes each. Neural network activity was recorded during the first five weeks of each culture’s in-vitro development. Stimulation sessions were delivered to each of the 59 electrodes. The False Discovery Rate technique was used to quantify the temporal evolution of dissociated cortical neurons. Our analysis focused on network responses that occurred within selected time window durations, namely 50 ms, 100 ms and 150 ms after stimulus onset.

**Results:**

Our results show an evolution in dissociated cortical neuronal network activity over time, that reflects the network synaptic evolution. Furthermore, we tested the sensitivity of our technique to different observation time windows and found that varying the time windows, allows us to capture different dynamics of the observed responses. In addition, when selecting a 150 ms observation time window, our findings indicate that cultures dissociated from the same brain tissue display trends in their temporal evolution that are more similar than those obtained from different brains.

**Conclusion:**

Our results emphasize that the FDR technique can be implemented without the need to make any particular assumptions about the data a priori. The proposed technique was able to capture the well-known dissociated cortical neuron networks’ temporal evolution, that has been previously observed in in-vivo and in intact brain tissue studies. Furthermore, our findings suggest that the time window that is used to capture the stimulus-evoked network responses is a critical parameter to analyze the electrical behavioral and temporal evolution of dissociated cortical neurons.

## Background

Culturing dissociated cortical neurons on Micro-Electrode Array (MEA) dishes is a powerful experimental tool for investigating functional and structural characteristics of in-vitro neuronal networks. Over the past few decades, MEAs have been frequently used to investigate the mechanisms that take place at the network level among cultured neurons and to answer fundamental questions regarding the cellular basis of learning, memory and synaptic developmental plasticity [1]. MEAs allow researchers to carry out long-term (up to a few months) non-invasive neural recordings using experimental setups that are easier to control and less complex than similar in-vivo systems. In general, MEA research falls in one of two categories: hybrid systems, in which artificial and natural intelligence are combined and biologically derived brain models that can be used to investigate how the brain works and how it forms structural and functional connections.

### Closed-loop experiments

MEA technology is often used to perform real-time experiments, also called closed-loop experiments, in which a feedback loop is implemented by delivering electrical stimuli to the electrodes while simultaneously recording from them. Electrical stimulation allows researchers to modulate neural activity in real-time in order to induce network plasticity or to simulate the effects of sensory input [2-5]. In [4,6-9] the authors investigated how to use electrical stimulation to evoke and modulate neural responses. For example, in [8] the authors demonstrated selective learning in a network of real cortical neurons by implementing a closed-loop stimulation protocol that allowed them to map evoked neuronal responses to known stimuli. These responses were used to generate learning curves that described how the repeated stimulation protocols were inducing changes in the synaptic connections of the neuronal network.

From this perspective, MEA technology can be used to investigate how living neurons could interact with artificial systems with the goal of building hybrid systems where artificial and natural intelligence coexist. These hybrid systems could also be used to simulate and study different pathological situations or neurological disorders, such as epilepsy and stroke [10]. For instance, MEA technology can be potentially used to investigate brain structures at the network level and to study the causes for most brain disorders such as Parkinson’s disease, Alzheimer’s and neuropathic pain.

### Brain modeling

Since the first studies of Ramón y Cajal [11] dating back over a century ago, researchers have been interested in investigating how neurons are physically connected in our nervous system and what these connections mean from a functional neuronal network perspective. To date, we know that functional connectivity modulates cognitive and behavioral states in the brain, but very little is known about functional networks and complex neuronal ensembles involving overlapping or multiple anatomical structures [12,13]. One limiting factor of this line of research is related to the fact that most studies require the use of expensive and ad-hoc imaging tools such special MRI systems combined with tightly controlled experiments and powerful image processing techniques [13].

In contrast, MEA recordings represent an innovative tool to build a simplified and yet realistic neuronal model able to simulate the functions and properties of brain layers [9]. Starting from simple brain layer models we can then increase the system complexity, trying to combine multiple layers and eventually building 3-D neuronal structures. Furthermore, MEA systems give researchers a greater flexibility in changing and readapting experimental setups as compared to the kinds of experiments that are currently used to draw maps of human functional connections [13].

Despite the innovative approaches and findings of the aforementioned studies, neuronal functional plasticity related to electrical stimulation (e.g. what happens when stimulating a neuronal culture during different phases of its in-vitro development) still needs to be fully investigated. For instance, very little is known about: (1) how a culture reacts if stimulated at different stages of development; (2) what are the mechanisms that allow such cultures to consistently respond to stimulation; (3) how important stimulation delivery is during early developmental stages; and (4) what are the real effects of stimulation on cultures stimulated repeatedly over time as compared to “never-stimulated” control cultures.

Even though some studies have investigated spontaneous electrical activity in dissociated cultures [14-17], many questions still need to be addressed before we can integrate these neuronal networks into larger and more complex systems. For instance, to our knowledge, there are no quantitative studies that assess how the development of dissociated rat cortical neurons can be affected by chronic external stimulation [3].

### Quantitative analysis of network activity and its temporal evolution

In this work, we aim to address two major limitations of the aforementioned research fields. Firstly, although hybrid neural-electrical circuits have been demonstrated, their functionality is inherently limited when the neuronal network is treated as a black box. An understanding of how those networks evolve with respect to specific stimuli (or lack thereof) will necessarily lead to hybrid systems with greater functionality and robustness. Our long-term goal is to understand how these systems respond to external stimulation, [18] how and why they vary their electrical activity over time [19,20].

Secondly, there is a lack of adequate statistical tools for processing and quantifying large spike-based data sets [21]. This deficit hinders investigators’ ability to identify significant changes in network connectivity amid populations of weakly tuned neurons with high spontaneous activity. As a result, the ultimate goal of exploring the relationship between neural circuit topology and behavior is compromised. Existing tools such as activity task neuroimaging are insufficiently sensitive both temporally and spatially [21].

Although various approaches have been presented for analyzing spike behavior in MEA recordings, these methods have tended to focus on raw statistical correlations without necessarily yielding meaningful insights into physiological network topology. In [22] the authors use Hidden Markov Models (HMMs) to estimate the number of states the neurons in the network can have. The authors assumed that neuronal networks only adopt three different firing patterns. This simplification was necessary to implement the HHM technique, but at the same time such an approach fails to capture the high variability and variety of neuronal network electrical responses.

Others [23] have proposed to use dynamic Bayesian networks to discover excitatory relationships in MEA recordings. In this work the authors were testing a computer algorithm capable of emphasizing the excitatory statistical connections in discrete-time networks. Their main assumption is that in the network only excitatory connections are important, while inhibitory connections are neglected. With respect to neuronal networks, such an assumption cannot be considered valid, thus their mathematical approach cannot capture the full complexity of live neuron interactions.

In both of these studies, the authors realize the importance of applying statistical techniques to identify sequences of firing neurons and find the functional network connectivity. However, despite the recognition of the relevance of statistical methods, there is a lack of literature investigating the physiological aspects of neuronal development [13,24].

We propose to use a well-known statistical technique, that has been proven successful in separating the non-null cases from the null cases in multiple hypothesis testing. For the first time, we aim to statistically quantify the temporal dynamics of dissociated cultured neuronal networks, without simplifying the underlying biological model. In this work, our goal was to address the above-mentioned issues by applying the False Discovery Rate (FDR) statistical analysis technique to MEA recordings and using its results to quantify biological and electro-physiological properties of dissociated neuronal networks during their first five weeks in-vitro. FDR identifies significant stimulus-response pairs among the numerous spontaneous spikes from the cultured neurons. Moreover, the FDR technique has been proven to be a valuable tool to overcome the traditional issues in multiple hypotheses testing problems, namely controlling the probability of erroneously rejecting even one of the true null hypotheses, otherwise known as the familywise error-rate (FWE) [25]. This allowed us to investigate the temporal evolution of cultured neural networks while presented with electrical stimulation during early development.

## Methods

The statistical analyses presented here were performed on neural spike data made available by Dr. Steve Potter in the Laboratory for Neuroengineering at Georgia Institute of Technology and Emory University School of Medicine. They comprise a series of MEA recordings from cultures of dissociated rat cortical neurons with bursting activity patterns, recorded over the first five weeks of their in-vitro development. Details of the cell culture methodology and electrophysiology can be found in [14].

To investigate network changes, we analyzed a large MEA data set composed of neuron spikes recorded from cultures of dissociated rat cortical neurons plated on MEA dishes with 59 recording electrodes each. There were 15 high-density high-volume (“dense”) cultures, as well as 7 high-density small-volume (“small”) and 6 low-density high-volume (“sparse”) ones. The culture density was chosen when plating the dissociated cortical neurons onto the MEAs, as described in [14]. Further details on different plating densities can be found in Table 1. Some neuron cultures were dissociated from the same original brain tissue; such cultures were defined as belonging to the same “batch” of brain tissue. The number of neuronal cultures dissociated from each batch is shown in Table 1.

**Table 1:** Different neuronal cultures

	**DENSE**	**SMALL**	**SPARSE**
Plating volume (*μ*L)	20	5	20
Density of suspension (cells/ *μ*L)	2500	2500	625
Nominal number of plated cells	50000	12500	12500
Number of studied cultures	15	7	6
Number of studied batches	6	2	2

To notice how the plating volume and density of suspension for dense and sparse cultures are the same, but they have a different number of plated cells. Small cultures have the same density of suspension as dense cultures, but the number of plated cells is the same as sparse cultures.

Neural network activity was recorded during the first five weeks of each culture’s in-vitro development. During this period, stimulation sessions (typically occurring daily) comprised of 50 electrical stimulus pulses were delivered to each of the 59 electrodes. These stimuli were delivered sequentially to every electrode on the MEA, once every 300 ms, while neural responses were recorded from all the other electrodes. Although it is well-known that neuronal network responses elicited during such stimulation sessions are complex and may last longer than 300 ms [26,27], we focused on network responses that occurred within selected time window durations, namely 50 ms, 100 ms and 150 ms after stimulus onset. This allowed us to account for three specific components of network responses known as 1) the network “direct responses” to stimulation, that are those occurring between 0 and 20 ms after stimulus (50 ms windows); 2) the “early post-synaptic spikes”, occurring 5–1000 ms after stimulus presentation (100 ms windows); 3) “Culture-wide barrages”, occurring at latencies greater than 100 ms (150 ms windows). These responses are thought to be the most representative of the stimulation effects [6-8,16,28,29].

The stimulus-evoked spike count was normalized by subtracting the average spontaneous spike count averaged over the chosen time window. The spontaneous spikes were recorded on the same experimental day as the stimulus-evoked spikes, from the same neural network. This technique allowed us to account for the natural variability in neuron firing activity that occurs as a result of axonal growth and network changes over time. The same stimulation protocol was delivered to every culture [14].

Each culture yielded a 59×59×50 data matrix (stimulated electrodes × recording electrodes × number of trials) of normalized spike counts on each day. We then averaged across trials to produce a 59×59 matrix of stimulus-response pairs (Z_*kj*_) per day per culture. These matrices were then interpreted for statistical significance (see Section “Statistical analysis”). Only those stimulus-response pairs determined to be statistically significant were used in the subsequent quantitative connectivity analysis.

In order to be able to quantify changes in the connectivity graphs with respect to time, we used two measures per experimental day: the average length of significant pairwise stimulus-response connections and the number of connections that every node displays. The former is a measure of how physically far the neurons can extend their connectivity pathways. The latter is a measure of how many significant connections every node can either generate or receive. In other words, this is a measure of how many significant hubs the network displays on any specific experimental day. We defined “supernodes” to be those nodes that display at least four significant connections, either incoming or outgoing. The existence of supernodes is consistent with the notion that biological networks tend to form ‘small-world’ networks, as previously showed in [30]. It is worth noting that the MEA electrode grid used in this work is directly connected to the underlying neuron network. However, given the limited number of electrodes and their size and spacing, this electrode grid cannot capture the full extent and complexity of the actual neuron connectivity. Consequently, every electrode (or node) is actually simultaneously recording from (and stimulating) multiple neurons (ranging from tens to hundreds). Considering the high neuronal connectivity, a single stimulus pulse is therefore potentially able to induce stimulus-evoked responses across the whole network either directly or through one or more synapses. As a result, when we identify connections and connectivity graphs, we are actually measuring connections between electrodes (nodes) and not single cells. Although it is not easy to quantify the exact number of neurons involved, it is reasonable to assume that each supernode connection comprises a number of main neurons ranging between 80 and 200.

### Statistical analysis

**Figure 1:**
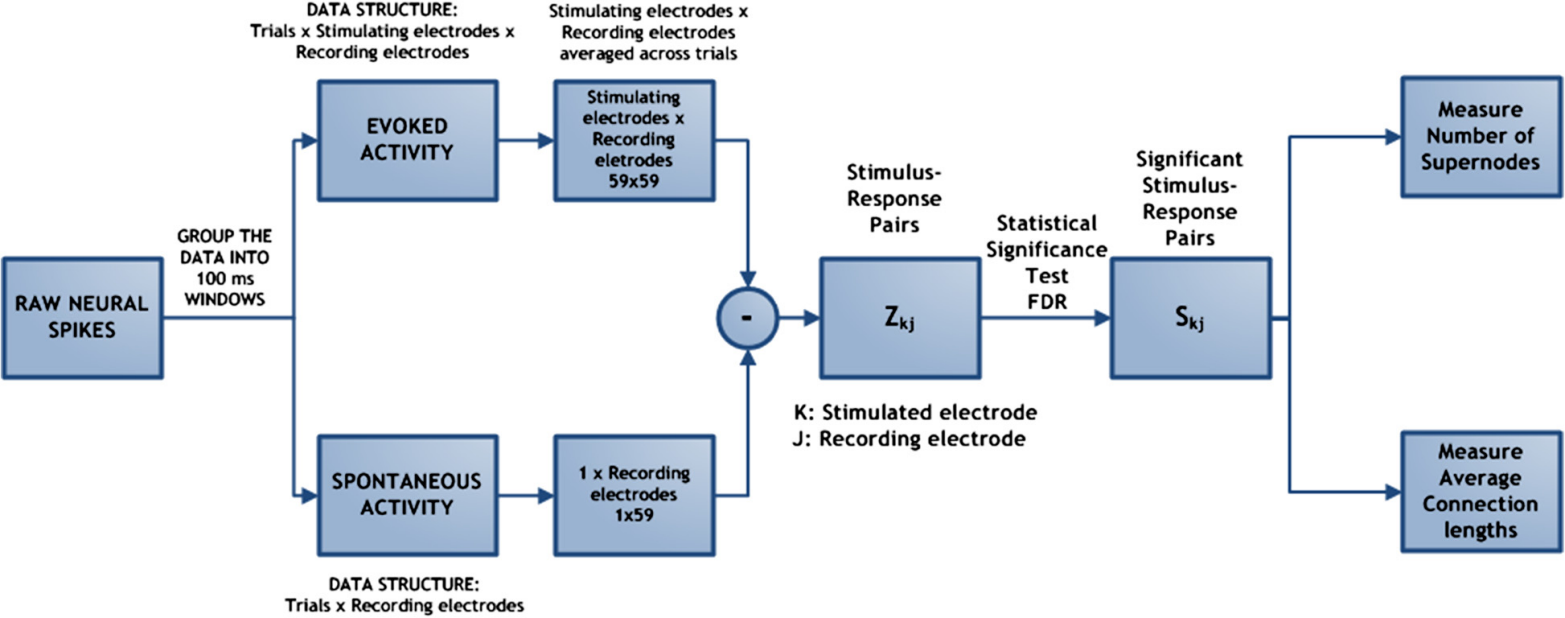
**Block Diagram of the implemented neuronal spike statistical analysis.** The raw neural spikes are divided into two groups, evoked activity and spontaneous activity, respectively stimulated and non-stimulated experimental sessions. Then the raw spikes were divided into time windows and averaged across repetitions. The average spontaneous activity was subtracted from the average evoked activity and fed into the False discovery Rate (FDR) statistical analysis technique. The output of the FDR is the significant stimulus-response pairs. Using these significant pairs we computed their average connection lengths and the number of supernodes.

In order to identify statistically significant stimulus-response pairs, we implemented the False Discovery Rate (FDR) analysis technique. The FDR technique is a multiple hypothesis testing procedure whose objective is to control the expected proportion of incorrectly rejected null hypotheses, as shown by Equation 1. We chose to use FDR because it has been proven to be effective when testing multiple hypotheses [31] in high dimensionality data sets. In our case the null hypothesis is that a given stimulus-response pair is not statistically significant. We applied the FDR to the average number of evoked spikes relative to the average number of spikes recorded when no stimulation was delivered, (*Z*_*kj*_) as shown in Figure 1.

### Statistical significance test: FDR

The False Discovery Rate is defined as:

(1) $$\text{FDR}=E\left[\frac{V}{R}\right]$$

where: 

- V is the number of false discoveries

- R is the total number of discoveries

FDR procedures are designed to control the expected proportion of incorrectly rejected null hypotheses, also called false discoveries V. In this work we chose FDR = 5%. The null hypothesis was defined as: H{0,kj} : While stimulating electrode k, electrode j does not respond.

Therefore, the FDR guarantees that no more than 5% of the stimulus-response pairs identified as being significant will actually be insignificant. The FDR was applied to each of the 59×59 elements of the matrix *Z*_*kj*_ (stimulus-response activity pairs, normalized by the network spontaneous activity) recorded for every experimental session and for every culture. The implemented mathematical analysis is shown in Figure 1.

Mathematically, the FDR technique defines the two hypotheses as follows:

(2) $$\begin{array}{l} H\{0,\text{kj}\}:Z_{\text{kj}}∼N(\mu_{0}=0,\sigma^{2})=f_{0}(Z_{\text{kj}})\\ H\{1,\text{kj}\}:Z_{\text{kj}}∼f_{1}(Z_{\text{kj}}) \end{array}$$

The statistic *T*_*kj*_, referred to as “local FDR” is then defined as:

(3) $$T_{\text{kj}}=\frac{\left(1-\varepsilon )f_{0}(Z_{\text{kj}}\right)}{\varepsilon f_{1}(Z_{\text{kj}})+(1-\varepsilon )f_{0}Z_{\text{kj}}}=\frac{\left(1-\varepsilon )f_{0}(Z_{\text{kj}}\right)}{f(Z_{\text{kj}})}$$

In other words, the local FDR function quantifies the relative likelihood of H0; values of *T*_*kj*_ close to 1 indicate a high likelihood of H0 whereas values closer to 0 indicate a low likelihood. The local FDR function is a measure of how similar the two distributions *f*_0_(*Z*_*kj*_) and *f*(*Z*_*kj*_) are, where *f*_0_(*Z*_*kj*_) is the null distribution density function and *f*(*Z*_*kj*_) is the alternative distribution function [31]. The parameter *ε* is called the non-null proportion [25,31] and represents the number of expected significant stimulus-response pairs. In our analysis we expect *ε* to always assume small values because we expect the number of significant connections to be lower than the number of possible pairwise connections in the network. The value of the parameter *ε* is estimated from the data before computing the local FDR functions [25]. If *T*_*kj*_ is close to 1, then the two distributions defined in the hypotheses are similar and the null hypothesis is selected. This indicates no significant relation between the stimulus, delivered to electrode k, and the neural response recorded at electrode j.

Next, the 59×59*T*_*kj*_’s are ranked from the smallest to the largest. The ordered local FDR functions are called *T*_1_,…,*T*_*p*_ where p=59×59. Significant local FDR functions are therefore *T*_*i*_, for *i*≤*k*, such that:

(4) $$k=\text{max}\left(I:\frac{\overset{k}{\underset{I=1}{\sum }}T_{I}}{k}\right)\leq \text{FDR}$$

This technique therefore guarantees that the average false positive rate over all significant stimulus-response pairs will be less than 5%.

## Results

**Figure 2:**
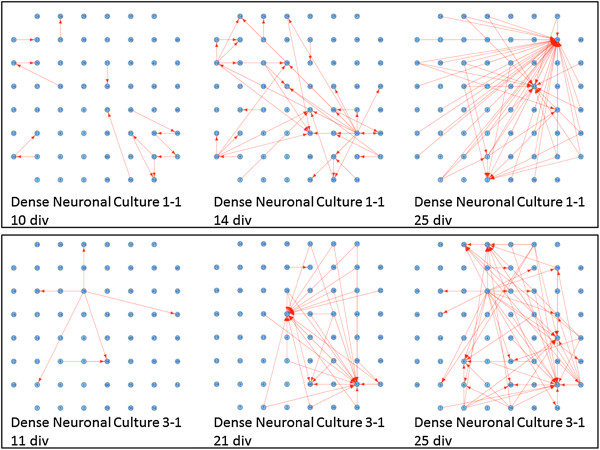
**Connectivity Graphs for dense neuronal cultures on different days after plating.** The top Panel shows connectivity graphs for culture 1-1, on days in-vitro (div) 10, 14 and 25. The bottom panel shows results for culture 3-1, on days in-vitro 11, 21 and 25.

Figure 2 shows typical connectivity graphs for two different cultures harvested from different brain tissues, on three separate days. Each red arrow indicates a statistically significant connection between a stimulated electrode and a recording one, as identified by the FDR analysis. Figure 2 suggests that neuronal connectivity tends to evolve over time, with increases in both the number of statistically significant stimulus/recording pairs as well as the average length of connections and the number of connections per active node.

**Figure 3:**
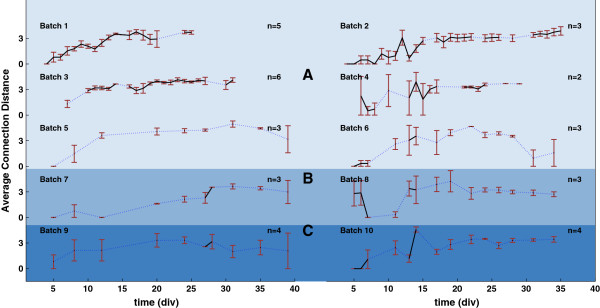
**Connection Lengths averaged across cultures harvested from the same tissue batch when using a 150 ms time window. A)** shows results derived from 6 dense culture batches. **B)** Results derived from 2 sparse density batches and **C)** displays results for 2 small density batches. Each panel shows results with respect to a different batch. The black solid line represents the average connection length within batches, while red vertical lines are the corresponding standard errors. Blue dotted lines represent missing experimental data points. n represents the population size for each batch.

**Figure 4:**
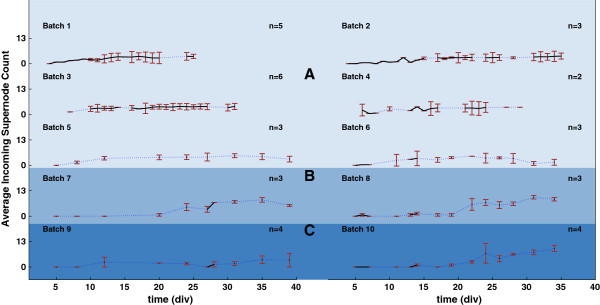
**Incoming supernode number averaged across different cultures harvested from the same tissue batch when using a 150 ms time window. A)** shows results for 6 dense culture batches. **B)** shows results for 2 sparse density culture batches and **C)** for 2 small density cultures. Black solid lines represent the average supernode number within batches, blue dotted lines represent missing experimental data points. Red vertical lines are the corresponding standard errors. n represents the population size for each batch.

**Figure 5:**
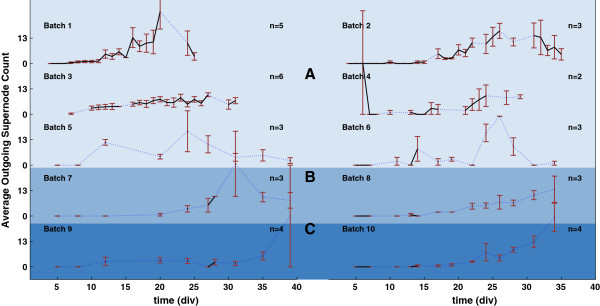
**Outgoing supernode number averaged across different cultures harvested from the same tissue batch when using a 150 ms time window. A)** shows results for 6 dense culture batches. **B)** shows results for 2 sparse density culture batches and **C)** for 2 small density cultures. Black solid lines represent the average supernode number within batches, blue dotted lines represent missing experimental data points and red vertical lines are the corresponding standard errors. n represents the population size for each batch.

In order to better analyze the changes in electrical activity versus time (and among different plating densities and neuron batches), we averaged the connection lengths and the supernode counts across cultures harvested from the same batch. The resulting graphs are shown in Figures 3, 4 and 5, where average connection distances, average incoming supernode count and outgoing supernode count are shown respectively for dense, small and sparse cultures when a 150 ms time window was chosen to record network activity. For every cell density, the number of cultures that we used to compute the average within batches is different and it is indicated in the figures with *n*. Due to the fact that recordings were not performed every day, we used a dotted blue line to indicate missing experimental days while a solid black line was used to plot the actual data sample means. The red error bars indicate the standard errors obtained when averaging cultures derived from the same batch. From these graphs, it is noticeable how the average connectivity pair lengths increase over time, then reach a plateau, following the expected network temporal evolution. Moreover, this behavior is observed to be consistent across cultures of different densities and across different time window durations (data not shown). An increase in average connection length means that stimulus-evoked responses are recorded from electrodes that are physically further from the stimulated electrode; evoked electrical activity is propagated more easily in the dish and over longer distances. The functional evolution in the studied neuronal networks reflect the natural temporal evolution of neural circuit formation. In fact, neural circuit formation occurs in three distinct stages: 1) Immature synapses form between axons and dendrites. 2) Synapses undergo maturation, which involves the conversion of silent synapses to active ones. 3) Excess synapses are eliminated or pruned to refine the neuronal connections within the circuit [10].

Figures 3, 4 and 5 reveal consistent neural development within batches, with more variable trends across batches. To quantify this, we ran one-way ANOVA tests on both connection lengths and number of supernodes within and across batches. Our results with respect to 50 ms and 150 ms windows suggest that the connection length variability within batches is not statistically significant (*p*≥0.05). On the contrary, cross-batch variability was statistically significant for connection lengths (*p* values for 50 ms and 150 ms windows were respectively *p*_50_=0.0207 and *p*_150_=0.0107). No significant variations were observed in both incoming (*p*_50_=0.357 and *p*_150_=0.204) and outgoing (*p*_50_=0.295 and *p*_150_=0.992) supernode counts. In contrast, variations observed within and across batches for a 100 ms observation window tested non-significant with p values for mean connection lengths, mean incoming and outgoing supernodes respectively equal to *p*_*length*_=0.673, *p*_*incoming*_=0.357 and *p*_*outgoing*_=0.295. We hypothesize that the observed variability in the analysis results is mainly due to the fact that when using different time window durations, one might or might not include in the analysis some of the phases of the network stimulus-evoked responses. In this view, our statistical analysis emphasizes the importance of the time window length selection and that common values that have been commonly utilized in previous works, such as 100 ms windows, might not yield optimal results.

**Figure 6:**
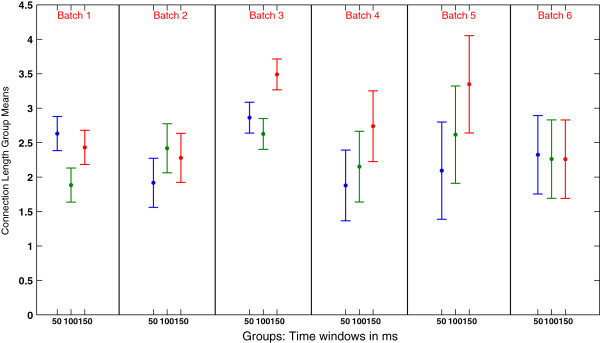
**Results of an ANOVA multiple comparison tests between the three time window group means for each batch.** The six panels show the three time window means (dot in the center of the lines) and 95% confidence intervals around them. When confidence intervals computed for different groups overlap, it means that such groups are not significantly different.

Furthermore, we tested the effects that different time windows had on the analysis results and whether the time window related changes were statistically significant. So we performed a one-way ANOVA test on the average connection lengths computed for the six dense batches in each time window. Then we performed an ANOVA multiple comparison test to identify which group means were statistically different from each other. The results of the multiple comparison test are shown in Figure 6, in which, the three group means and their 95% confidence intervals for every dense batch are shown. In batch 1 and batch 3, there are group means significantly different from the others. In batch 1 the second group mean (100 ms window) is significantly different from the other two group means, while in batch 3 is the third group mean to be significantly different from the others. It can be seen how differences between results generated using different time windows are significant in batch 1, where the 100 ms group mean is significantly different from the other two (*p*=7.051×10^-^4) and in batch 3 where 150 ms group mean is significantly different (*p*=8.126×10^-^6). This implies that different time windows generate significantly different results in two batches out of six. On the contrary, differences between the three group means are non-significant for the remaining batches.

## Discussion

This work has adapted a statistical technique for identifying significant neuronal connectivity between pairs of electrodes in a micro-electrode array dish. This work has furthermore developed two metrics for describing connectivity in the MEA dish: (1) the average distance between stimulus and recording electrodes, and (2) the existence of “supernode” electrodes, which form functional hubs connecting to a large number of other electrodes. Finally, this work has used these metrics to quantify connectivity trends in MEA cultures of dissociated rat cortical neurons, including culture preparations characterized as “Dense”, “Sparse” and “Small”. In all cases, the MEA dishes showed two phases of development with respect to neuronal connectivity over a period of about 40 days. The first phase was characterized by relatively little significant neuronal connectivity within the MEA dish, this phase typically lasted five days. The second phase, lasting 10-15 days, is characterized by a rapid growth in the sophistication of network connectivity, both in terms of average connection length and number of supernodes. At the end of the second phase, network growth tends to plateau.

It is interesting to notice that towards the end of the experiments (35 div) our statistical results show that the number of significant connections begins to decrease in some batches. This might be caused for several reasons including changes in neuron density, glial cell proliferation or the fact that the networks might become less sensitive to stimulation over time. We observed that after 35 div the spontaneous firing rate can start to decrease and stimulus evoked responses decrease accordingly. This is in agreement with what found in [14], where the authors found that after one month in vitro, the network’s overall firing rate was lower while its bursting activity increased.

Furthermore, our findings for 50 ms and 150 ms time windows, suggest that the observed neuronal networks display similar behavioral trends within neuron cultures derived from the same brain tissue with non-significant variations in their connection lengths. On the contrary, temporal evolution seems to display statistically significant differences when analyzing cultures harvested from different brain tissues, as quantified by the ANOVA test results (*p* value for 50 ms windows is *p*_50_=0.0207, and *p* value for 100 ms windows is *p*_150_=0.0107).

Two plausible explanations can justify the observed behaviors: 1) Cultures derived from the same brain tissues were grown, fed and recorded from at the same time and exposed to the same experimental conditions. On the contrary, experimental conditions might have been slightly different for cultures derived from different batches because they were grown during different periods of time. In this view the different experimental conditions could explain the high variability across batches. 2) Despite these neurons having been dissociated before plating, they could still retain some innate characteristics and properties originating from the brain tissue they were derived from. While the former explanation is more plausible, considering neuron sensitivity to experimental conditions. The latter is intriguing because it suggests that dissociated neurons retain essential properties of the original brain cortical tissue they were harvested from. If so, then electrical activity may be determined by genetic factors to a much larger extent than previously thought. Further investigation is warranted.

In previous studies, the gold standard to quantify the electrical activity of neuronal networks cultured on MEA dishes was to measure the overall network activity by summing the number of spikes detected per unit time over all electrodes [14]. Although this metric has proven beneficial when assessing the total network activity or network bursting activity, it is not specific or accurate enough to quantify the networks’ temporal evolution. Furthermore, given the randomness and variability associated with the spontaneous activity of such networks, it also lacks the statistical features that are valuable to minimize the effects of randomness in MEA recording results. Our findings suggest that FDR analysis is a valuable technique to investigate and quantify dissociated cortical networks’ temporal evolution when combined with more physiological metrics that can track changes in network activity.

One last consideration regarding the statistically significant connectivity graphs that are the results of the FDR analysis. It is important to notice that the identified connections are not necessarily direct connections between two nodes, but they can hide intermediate hops and more complex activity patterns. This issue gets even more complex if we keep in mind that the electrode connections are an overall and over-simplified representation of the neuron network connectivity. Unfortunately, with this kind of MEA dishes it is arduous to track the real neuronal connections that underlie electrode activation.

Despite the results presented in this work, further studies will be necessary to understand the role of chronic external stimulation in dissociated cortical neuron development. Specifically, while this work identifies characteristic phases of MEA network development, it is not known whether those changes are occurring spontaneously or in response to the daily stimulation protocol. Further investigation is needed in which the neuronal connectivity of unstimulated MEA arrays is compared to that of chronically stimulated ones. Preliminary evidence [18] suggests that electrical activity may shape network functional properties.

Our findings are consistent with previous results in the literature. For instance in [32], the authors have investigated the presence and the importance of “brain hubs” in functional brain organization. These brain hubs play a key role in global information integration between different parts of the brain connections.

In the future, we will develop this work by investigating the specific role of electrical stimulation in regulating neuronal development. Specifically, we will implement associative learning protocols in MEA dishes such as those described in [33]. Protocols will use two different sets of external electrical stimuli. The Unconditional Stimulus (US) will be chosen from those stimuli that do not produce any evoked network response, whereas the Conditional Stimulus (CS) will be chosen from among those stimuli that produce a distinctive network activity. By comparing the network responses to the different stimuli and characterizing their temporal evolution, we will be able to study in greater detail the learning processes that take place in dissociated cortical neurons. Furthermore, in order to improve the significance of our analytical approach, the methods introduced in this work could be applied to synthetic data following the approach presented in [34] and this will be the subject of a future study and publication.

## Conclusions

We studied how dissociated cortical neurons respond to chronic electrical stimulation. In particular we investigated the temporal evolution of neuronal activity in response to a constant electrical stimulation protocol over the first 5 weeks of neuronal development. Our goal was to quantify changes in neuronal network connectivity, in dissociated cortical neurons using statistical analysis. We hypothesized that both external stimuli and network functional evolution were fundamental in neuronal development as previously shown in the literature. In fact, our results show an evolution in network activity in two ways. Neuronal connectivity tends to evolve over time, with changes in both the number of statistically significant stimulus/recording pairs as well as the average length of connections and the number of connections per active node. We therefore propose that the FDR analysis combined with two metrics, the average connection length and the number of highly connected “supernodes” are meaningful techniques for describing neuronal connectivity in MEA dishes. Furthermore, our results indicate that when analyzing stimulus-evoked responses recorded within 50 ms and 150 ms time windows from stimulus onset, cultures dissociated from the same brain tissue display trends in their temporal evolution that are more similar than those obtained with respect to different batches, as quantified by the statistical tests within and across batches. We suggest two hypotheses that could help explain the observed phenomena: 1) Cultures derived from the same brain tissues were cultured and exposed to experiments in the same time periods and under very similar experimental conditions, this could have induced the similarities in the observed results. 2) Our findings could indicate that even after dissociation, these neurons preserved some of the properties and characteristics of the original brain tissue they were harvested from. This would indicate that genetic information and genetic programs control neural development and neural firing more than previously hypothesized [19].

## Authors’ contributions

AN performed the statistical analyses, interpreted the results, and wrote the paper. JX assisted with the statistical analysis. IO assisted with interpreting the results and writing the paper. All authors read and approved the final manuscript.
